# Supplementary material for: The causal role of circulating inflammatory markers in osteoporosis: a bidirectional Mendelian randomized study
Source: Front Immunol. 2024 Jul 18;15:1412298. doi: 10.3389/fimmu.2024.1412298 (PMC11291241; doi:10.3389/fimmu.2024.1412298)
Supplement: Supplementary file 1 [file DataSheet_1.zip › supplementary material/OSTEOPOROSIS-CIM/GCST90274775.tsv.gz_forest.pdf]

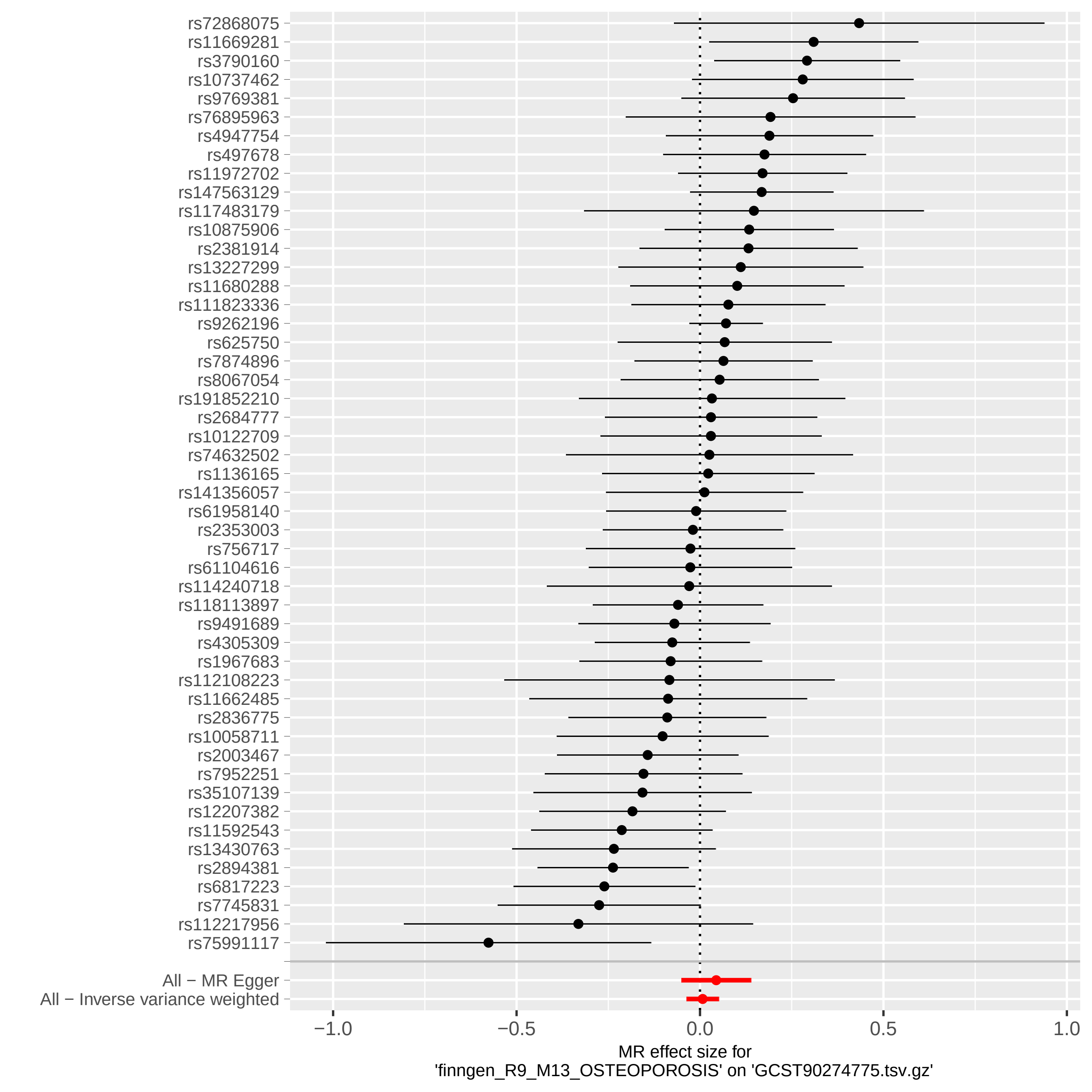
rs72868075
rs11669281
rs3790160
rs10737462
rs9769381
rs76895963
rs4947754
rs497678
rs11972702
rs147563129
rs117483179
rs10875906
rs2381914
rs13227299
rs11680288
rs111823336
rs9262196
rs625750
rs7874896
rs8067054
rs191852210
rs2684777
rs10122709
rs74632502
rs1136165
rs141356057
rs61958140
rs2353003
rs756717
rs61104616
rs114240718
rs118113897
rs9491689
rs4305309
rs1967683
rs112108223
rs11662485
rs2836775
rs10058711
rs2003467
rs7952251
rs35107139
rs12207382
rs11592543
rs13430763
rs2894381
rs6817223
rs7745831
rs112217956
rs75991117
All − MR Egger
All − Inverse variance weighted
−1.0
−0.5
0.0
0.5
1.0
MR effect size for
'finngen_R9_M13_OSTEOPOROSIS' on 'GCST90274775.tsv.gz'
